# Supplementary material for: Prevalence of Chronic Progressive Lymphedema in the Rhenish German Draught Horse
Source: Animals (Basel). 2023 Mar 9;13(6):999. doi: 10.3390/ani13060999 (PMC10044062; doi:10.3390/ani13060999)
Supplement: Supplementary file 1 [file animals-13-00999-s001.zip › Supplementary-FiguresS1-S7-TableS1-TableS17.pdf]

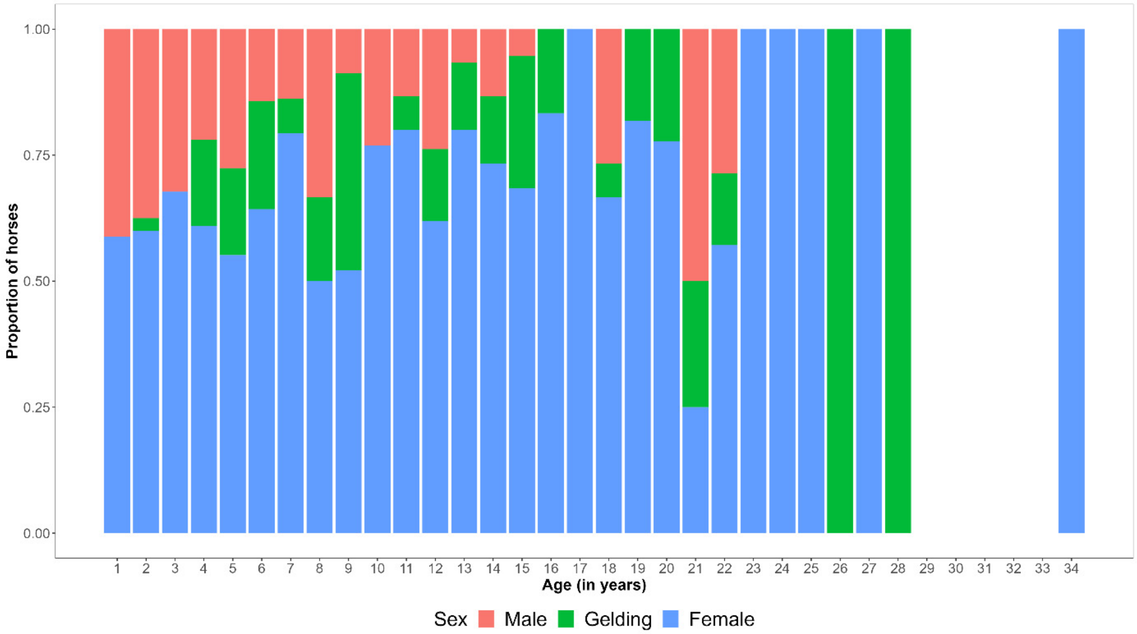

Figure S1. Proportional distribution of the 493 sampled Rhenish German draught horses by sex (male, gelding and female) and age in years.

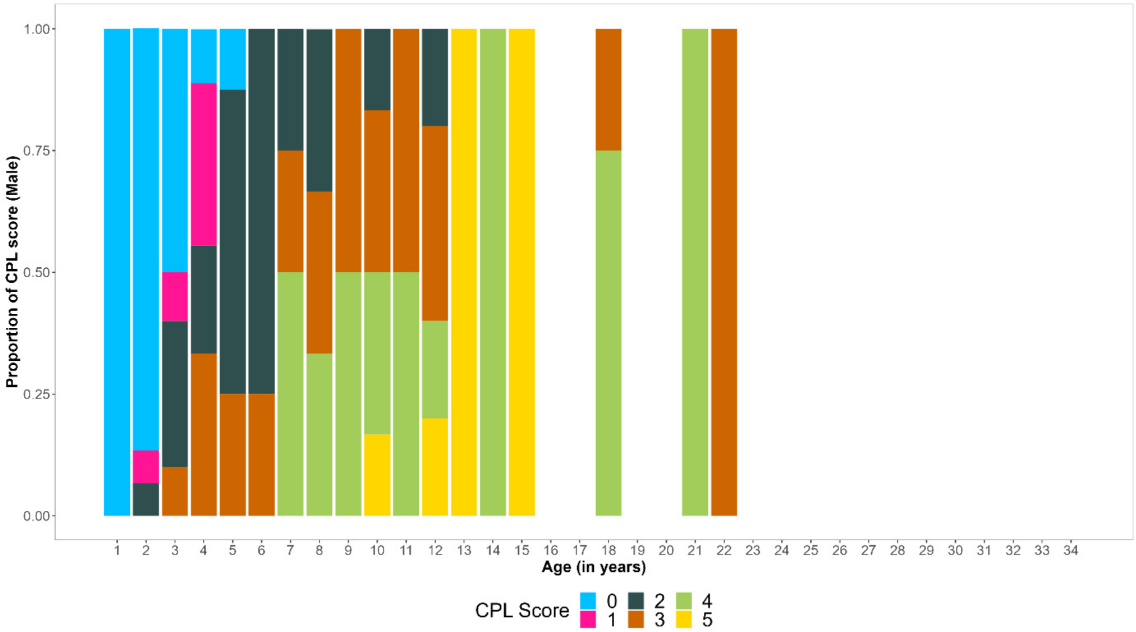

Figure S2. Proportional distribution of the 111 sampled male Rhenish German draught horses by scores of chronic progressive lymphedema (CPL) and age in years.

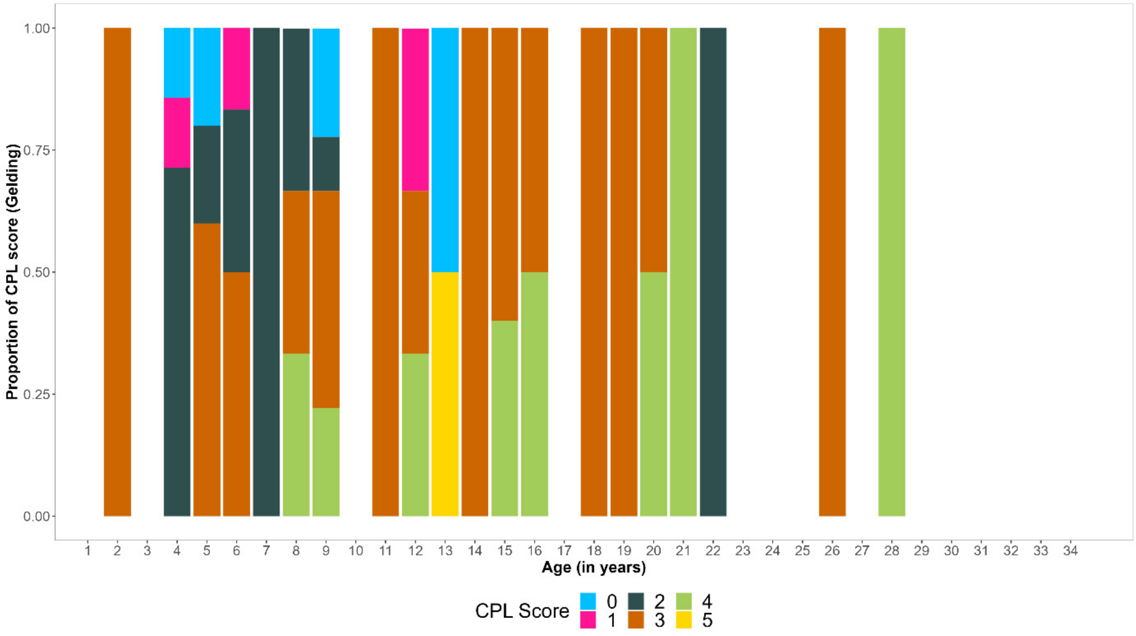

Figure S3. Proportional distribution of the 57 sampled Rhenish German draught horses (geldings) by scores of chronic progressive lymphedema (CPL) and age in years.

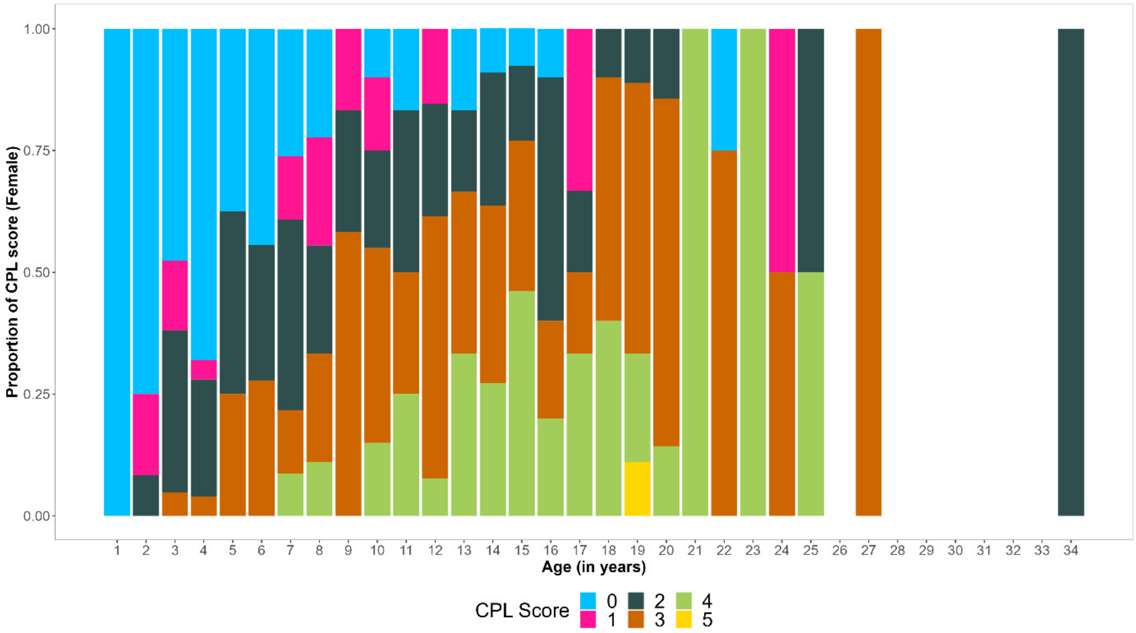

Figure S4. Proportional distribution of the 325 sampled female Rhenish German draught horses by scores of chronic progressive lymphedema (CPL) and age in years.

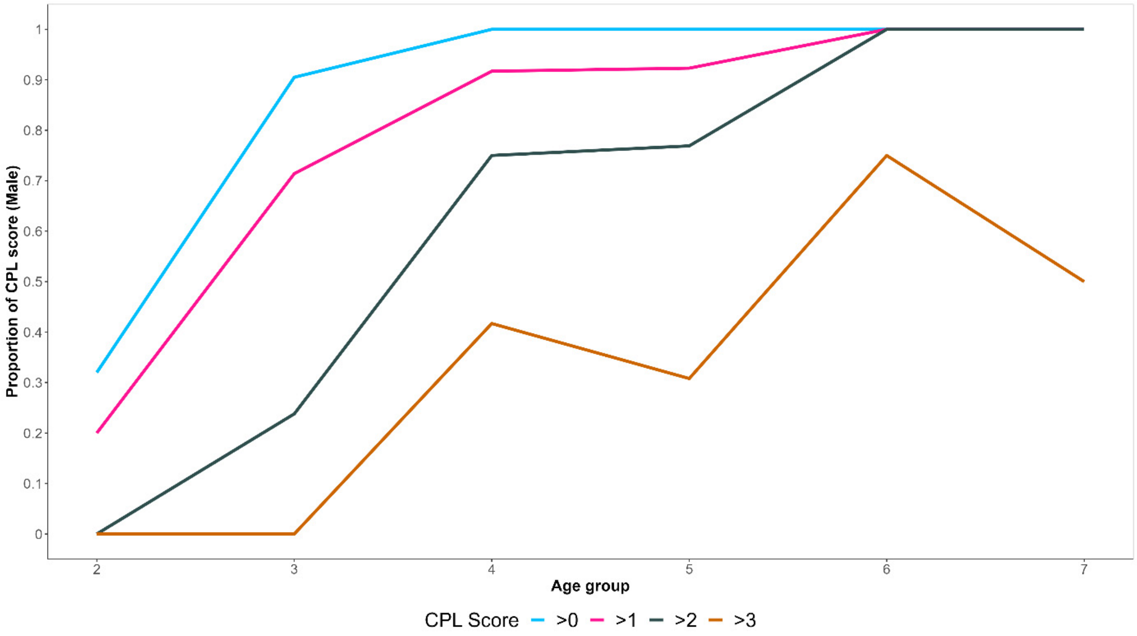

Figure S5. Distribution of scores >0, >1, >2, and >3 for chronic progressive lymphedema (CPL) by sex and age groups of the 111 male Rhenish German draught horses. Age group 2 contains horses aged 1-2 years, age group 3 horses aged 3-5 years, age group 4 horses aged 6-8 years, age group 5 horses aged 9-11 years, age group 6 horses aged 12-17 years, and age group 7 horses aged 18 or more years. The blue line represents the proportion of male horses in the respective age group with CPL-scores 1-5, the red line the proportion with CPL-scores 2-5, the dark green line the proportion with CPL-scores 3-5, and the brown line the proportion with CPL-scores 4-5.

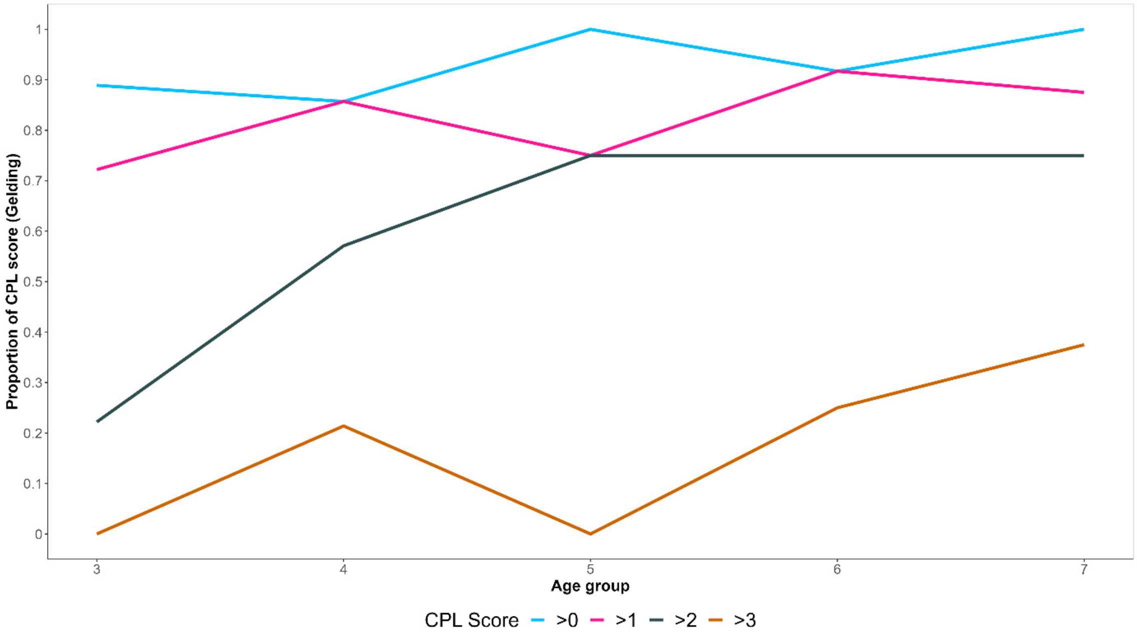

Figure S6. Distribution of scores >0, >1, >2, and >3 for chronic progressive lymphedema (CPL) by sex and age groups of the 57 Rhenish German draught horse geldings. Age group 2 contains horses aged 1-2 years, age group 3 horses aged 3-5 years, age group 4 horses aged 6-8 years, age group 5 horses aged 9-11 years, age group 6 horses aged 12-17 years, and age group 7 horses aged 18 or more years. The blue line represents the proportion of geldings in the respective age group with CPL-scores 1-5, the red line the proportion with CPL-scores 2-5, the dark green line the proportion with CPL-scores 3-5, and the brown line the proportion with CPL-scores 4-5.

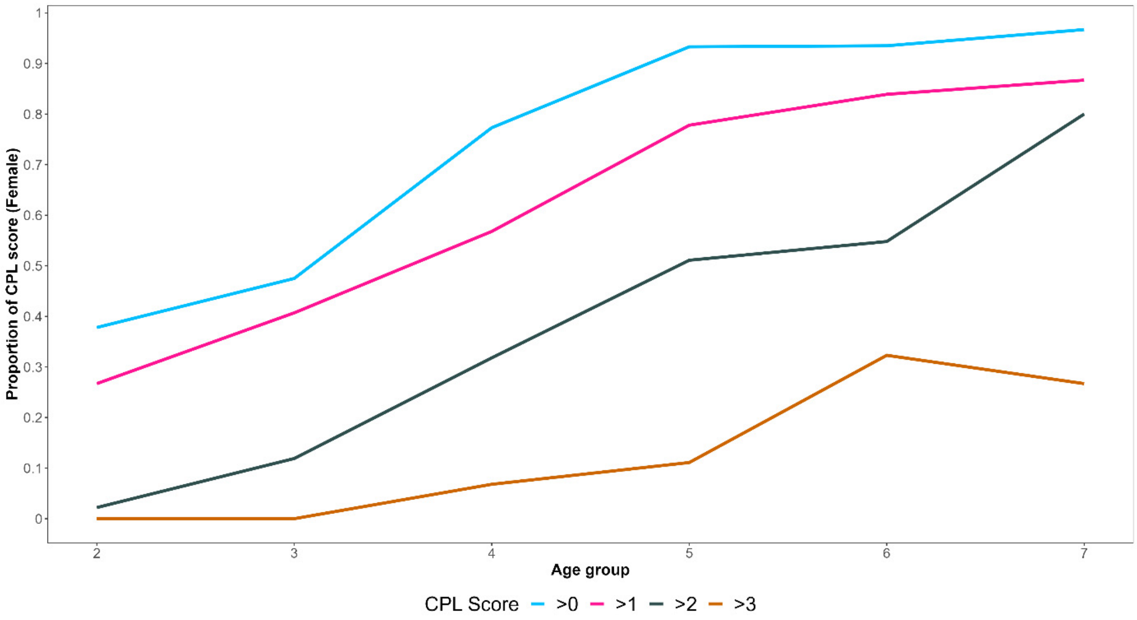

Figure S7. Distribution of scores >0, >1, >2, and >3 for chronic progressive lymphedema (CPL) by sex and age groups of the 325 female Rhenish German draught horses. Age group 2 contains horses aged 1-2 years, age group 3 horses aged 3-5 years, age group 4 horses aged 6-8 years, age group 5 horses aged 9-11 years, age group 6 horses aged 12-17 years, and age group 7 horses aged 18 or more years. The blue line represents the proportion of female horses in the respective age group with CPL-scores 1-5, the red line the proportion with CPL-scores 2-5, the dark green line the proportion with CPL-scores 3-5, and the brown line the proportion with CPL-scores 4-5.

Table S1. Questionnaire on horse-farm related variables including housing types, feeding and management.

Farm-related factor	Category	Number of horses	Percentage
**Stable type**			
Box stall	1	204	41.38
Loose barns with boxes	2	149	30.22
Open stable	3	117	23.73
Pasture	4	13	2.64
Unknown	5	10	2.03
**Bedding type**			
Barley straw	1	57	11.56
Rye straw	2	16	3.25
Wheat straw	3	127	25.76
Wood shavings	4	12	2.43
Barley + wheat straw	5	167	33.87
Rye + wheat straw	6	55	11.16
Any straw	7	28	5.68
Unknown	8	31	6.29
**Time interval for cleaning out the stable**			
1-7 days	1	254	51.52
2-4 weeks	2	145	29.41
1-2 months	3	63	12.78
>2 months	4	31	6.29
**Outdoor access in winter**			
Paddock	1	142	28.80
Pasture	2	341	69.17
Unknown	3	10	2.03
**Average hours per day of outdoor exercise in autumn and winter**			
24 h	1	115	23.33
2-8 h	2	368	74.65
Unknown	3	10	2.03
**Outdoor access in summer**			
Paddock	1	34	6.90
Pasture	2	357	72.41
Pasture and paddock	3	92	18.66
Unknown	4	10	2.03
**Type of roughage fed in winter months**			
Hay	1	198	40.16

Hay + straw	2	89	18.05
Hay + silage or haysilage	3	153	31.03
Haysilage	4	39	7.91
Unknown	5	14	2.84
**Type of concentrates fed in winter months**			
Oats or oats and pellets or oats and barley or oats and muesli or oats and pellets	1	240	48.68
Muesli, Muesli + Pellets	2	53	10.75
Pellets, barley, cornflakes	3	74	15.01
No concentrates	4	116	23.53
Unknown	5	10	2.03
**Type of concentrates fed in summer months**			
Oats or oats and pellets or oats and barley or oats and muesli or oats and pellets	1	105	21.30
Muesli, Muesli + Pellets	2	34	6.90
Pellets, barley, cornflakes	3	28	5.68
No concentrates	4	313	63.49
Unknown	5	13	2.64
**Mineral feed**			
No	1	325	65.92
Yes	2	144	29.21
Unknown	3	24	4.87
**Length of hoof trimming intervals**			
4–8 weeks	1	147	29.82
9–12 weeks	2	105	21.30
13–16 weeks	3	85	17.24
>16 weeks	4	26	5.27
Unknown	5	130	26.37
**Type of hoof care**			
Cutting of hoof horn	1	365	74.04
Shoeing	2	91	18.46
Unknown	3	37	7.51
**Type of use or work applications for horses**			
Wagon rides	1	203	41.18
Riding	2	26	5.27
Breeding	3	65	13.18
Use in agriculture or milk production	4	65	13.18
Unknown	5	134	27.18
**Daily hours of work with horses**			
1-2 h /day	1	45	9.13
2-4 h/day	2	66	13.39
Unknown	3	382	77.48
**Days per week of work with**			

horses			
1-2 days/3-4 weeks	1	10	2.03
1-2 days/week	2	58	11.76
3-4 days/week	3	37	7.51
4-7 days/week	4	53	10.75
Unknown	5	335	67.95

Table S1. Scoring system for clinical evaluation of chronic progressive lymphedema based on Wallraf [25], Affolter [3] and de Keyser et al. [2].

CPL-Score	Clinical Signs	Localization
**0**	No changes	
**1**	Scaling	Below the fetlock joint, medial, lateral, dorsal, palmar/plantar
**2**	Hyperaemia<br>Scaling<br><br>Initial hyperkeratosis<br>Slight skin thickening	Below the fetlock joint or up to fetlock, medial, lateral, dorsal, palmar/plantar
**3**	Moistened hair patches<br>Exudation<br>Hair dull and erect<br>Hyperkeratosis<br>Moderate skin thickening<br>Initial skin fold	Up to fetlock or above fetlock, medial, lateral, dorsal, palmar/plantar
**4**	Bleeding wounds<br>Ulceration and exudation<br>Hair dull and erect<br>Severe hyperkeratosis<br>Severe skin thickening<br>Multiple skin folds<br>Multiple skin nodules	Above fetlock, medial, lateral, dorsal, palmar/plantar
**5**	Greasy, foul-smelling skin patches<br>Bleeding wounds<br>Severe Hyperkeratosis<br>Severe skin thickening<br>Multiple skin folds<br>Multiple skin nodules<br>Severe mechanical disturbance, lameness<br>Reduced general condition possible	Above fetlock, medial, lateral, dorsal, palmar/plantar

Table S3. Distribution of coat colours by CPL-scores in Rhenish German draught horses.

Coat colour	No of horses	Percentage of horses	CPL-Score						
			0	1	2	3	4	5	Mean ± SD
Chestnut	178	36.1	37.0	4.5	21.9	23.6	12.4	0.6	1.713±1.496
-without roan	163	33.0	38.7	4.3	20.9	23.9	11.7	0.6	1.675±1.503
-with roan	15	3.0	20.0	6.7	33.3	20.0	20.0	0	2.133±1.407
Black	42	8.5	35.7	16.7	19.1	19.1	9.5	0	1.500±1.401
-without roan	27	5.5	37.0	22.2	18.5	11.1	11.1	0	1.370±1.391
-with roan	15	3.0	33.3	6.7	20.0	33.3	6.7	0	1.733±1.438
Bay	273	55.4	32.6	5.9	19.8	25.6	14.3	1.8	1.886±1.533
-without roan	212	43.0	31.1	5.7	21.7	27.4	13.2	0.9	1.887±1.479
-with roan	61	12.4	37.7	6.6	13.1	19.7	18.0	4.9	1.886±1.723
Total	493	100	170	31	101	120	65	6	1.696±1.464

Table S4. Results of the generalized linear mixed model 1 with degrees of freedom (DF), F-values and *p*-values for CPL-max in Rhenish German draught horses.

Source of variation	DF	Normal distribution F-value	*p*-Value	Multinomial distribution F-value	*p*-Value
Breeding association	3	20.21	<0.0001	21.12	<0.0001
Sex	2	6.01	0.0027	5.88	0.0030
Coat color	2	3.33	0.0367	5.05	0.0068
Age by sex linear	3	100.06	<0.0001	58.66	<0.0001
Age by sex quadratic	3	37.46	<0.0001	30.83	<0.0001

Table S5. Results of the generalized linear mixed model 1 with degrees of freedom (DF), F-values and *p*-values for CPL-sum in Rhenish German draught horses.

Source of variation	DF	Normal distribution		Multinomial distribution	
		F-value	*p*-Value	F-value	*p*-Value
Breeding association	3	18.52	<0.0001	19.26	<0.0001
Sex	2	3.26	0.0395	5.56	0.0041
Coat color	2	2.90	0.0562	4.85	0.0083
Age within sex linear	3	79.33	<0.0001	59.62	<0.0001
Age within sex quadratic	3	26.91	<0.0001	29.36	<0.0001

Table S6. Results of the generalized linear mixed model 1 (binomial distribution with logit link function) with degrees of freedom (DF), F-values and *p*-values for CPL-score >0, CPL-score >1, and CPL-score >2, and CPL-score >3 in Rhenish German draught horses.

Source of variation	DF	F-values for CPL-scores				*p*-Values for CPL-scores			
		>0	>1	>2	>3	>0	>1	>2	>3
Breeding association	3	7.05	9.19	11.93	4.51	0.0001	<0.0001	<0.0001	0.0039
Sex	2	4.82	4.27	0.98	0.50	0.0085	0.0145	0.3750	0.6090
Coat color	2	1.23	1.19	2.17	3.18	0.2940	0.3041	0.1156	0.0426
Age within sex linear	3	22.37	23.00	15.13	7.53	<0.0001	<0.0001	<0.0001	<0.0001
Age within sex quadratic	3	6.98	11.01	6.10	4.62	<0.0001	<0.0001	0.0004	0.0034

Table S7. Results of the generalized linear mixed model 1 (multinomial distribution with cumulative logit link function) with degrees of freedom (DF), F-values and *p*-values for CPL-scores of horses aged ≥1 year, ≥2 years, ≥3 years, and ≥4 years in Rhenish German draught horses.

Source of variation	DF	F-values for horses aged (years)				*p*-Values for horses aged (years)			
		≥1	≥2	≥3	≥4	≥1	≥2	≥3	≥4
Breeding association	3	21.83	21.25	19.89	18.45	<0.0001	<0.0001	<0.0001	<0.0001
Sex	2	4.17	2.10	1.81	1.70	0.0162	0.1242	0.1645	0.1847
Coat color	2	4.76	3.83	4.35	3.36	0.0090	0.0225	0.0136	0.0360
Age within sex linear	3	42.79	30.64	24.70	15.77	<0.0001	<0.0001	<0.0001	<0.0001

Age within sex quadratic	3	21.86	15.86	13.63	9.33	<0.0001	<0.0001	0.0004	0.0034

Table S8. Results of the generalized linear mixed model 1 (multinomial distribution with cumulative logit link function) with degrees of freedom (DF), F-values and *p*-values for CPL-scores of horses aged ≥6 year, ≥8 years, ≥10 years, and ≥12 years in Rhenish German draught horses.

**Source of variation**	**DF**	**F-values for horses aged (years)**				***p*-Values for horses aged (years)**			
		**≥6**	**≥8**	**≥10**	**≥12**	**≥6**	**≥8**	**≥10**	**≥12**
Breeding association	3	13.69	8.99	4.14	3.81	<0.0001	<0.0001	0.0076	0.0123
Sex	2	0.24	0.49	1.35	1.26	0.7832	0.6157	0.2629	0.2868
Coat color	2	3.45	1.59	0.71	1.04	0.0333	0.2063	0.4951	0.3582
Age within sex linear	3	9.08	4.12	3.02	1.02	<0.0001	0.0074	0.0318	0.3883
Age within sex quadratic	3	6.22	3.27	2.89	0.96	0.0004	0.0224	0.0376	0.4160

Table S9. Estimates of odds ratios (ORs) with their 95% confidence intervals (95-CI) for age by sex effects and the *p*-values for regression coefficients using the generalized linear mixed model 1 (multinomial distribution with cumulative logit link function) for CPL-scores of horses aged ≥1, ≥2, ≥3, ≥4, ≥5, ≥6, ≥7, ≥8, ≥9, ≥10, ≥11, ≥12, ≥13, ≥14, and ≥15 years in Rhenish German draught horses. Given are the ORs for an increase by one year compared to the respective mean age in years of horses per age group.

**Age group – Mean age**	**ORs (95-CI)**<br>***p*-Values for linear and quadratic regression**		
	Male	Gelding	Female
All horses (n=493)			
7.8	1.845 (1.667-2.042)	1.247 (1.090-1.426)	1.442 (1.365-1.524)
	<.0001<br><.0001	0.0563<br>0.4018	<.0001<br><.0001
≥ 1 year (n=425)			
8.9	1.603 (1.457-1.764)	1.227 (1.095-1.374)	1.356 (1.289-1.426)
	<.0001<br><.0001	0.0638<br>0.4285	<.0001<br><.0001
≥ 2 years (n=385)			
9.7	1.492 (1.353-1.644)	1.236 (1.112-1.374)	1.301 (1.236-1.369)
	<.0001<br><.0001	0.0392<br>0.3088	<.0001<br><.0001
≥ 3 years (n=354)			
10.4	1.408 (1.270-1.561)	1.229 (1.115-1.354)	1.291 (1.223-1.363)
	<.0001<br><.0001	0.0336<br>0.2794	<.0001<br><.0001

≥ 4 years (n=313)			
11.3	1.360 (1.213-1.524)	1.183 (1.066-1.313	1.224 (1.156-1.295)
	<.0001	0.1782	<.0001
	0.0018	0.5721	<.0001
≥ 5 years (n=284)			
12.0	1.330 (1.165-1.519)	1.170 (1.046-1.310)	1.204 (1.134-1.277)
	0.0007	0.2516	<.0001
	0.0041	0.6049	<.0001
≥ 6 years (n=256)			
12.7	1.229 (1.067-1.416)	1.211 (1.054-1.392)	1.178 (1.106-1.254)
	0.0119	0.1730	<.0001
	0.0273	0.4054	0.0003
≥ 7 years (n=227)			
13.5	1.163 (1.003-1.348)	1.175 (1.018-1.355)	1.133 (1.055-1.218
	0.0203	0.2727	0.0010
	0.0355	0.4964	0.0041
≥ 8 years (n=209)			
14.0	1.142 (0.949-1.374)	1.182 (1.015-1.376)	1.108 (1.029-1.193)
	0.0483	0.2810	0.0067
	0.0581	0.4929	0.0167
≥ 9 years (n=186)			
14.7	1.056 (0.875-1.273)	1.135 (0.866-1.488)	1.083 (1.000-1.174)
	0.0843	0.7129	0.8471
	0.0901	0.8471	0.0422
≥ 10 years (n=160)			
15.5	1.021 (0.813-1.283)	1.115 (0.884-1.407)	1.077 (0.979-1.185)
	0.0269	0.6456	0.0446
	0.0274	0.7469	0.0540
≥ 11 years (n=145)			
16.1	0.937 (0.722-1.215)	1.108 (0.860-1.428)	1.081 (0.970-1.206)
	0.0496	0.7371	0.0530
	0.0467	0.8265	0.0589

≥ 12 years (n=124)			
16.9	0.600 (0.385-0.935)	1.055 (0.768-1.450)	1.036 (0.916-1.170)
	0.2448	0.9686	0.1962
	0.3075	0.9867	0.1788
≥ 13 years (n=109)			
17.5	0.640 (0.381-1.075)	1.041 (0.777-1.393)	1.019 (0.887-1.171)
	0.2448	0.8914	0.2436
	0.3595	0.8466	0.2045
≥ 14 years (n=94)			
18.1	0.717 (0.357-1.438)	0.939 (0.692-1.275)	1.040 (0.888-1.219)
	0.3994	0.4112	0.2265
	0.4350	0.4040	0.2030
≥ 15 years (n=75)			
19.1	0.686 (0.275-1.713)	0.601 (0.331-1.091)	1.133 (0.925-1.388)
	0.2005	0.0545	0.0943
	0.1985	0.0572	0.0974
≥ 16 years (n=63)			
19.8	0.251 (0.029-2.146)	0.575 (0.228-1.447)	0.964 (0.752-1.236)
	0.1528	0.1103	0.9900
	0.1529	0.1045	0.9383

Table S10. Results of the generalized linear mixed model for each one-horse farm-related factor added to model 1 with degrees of freedom (DF), F-values and *p*-values for CPL-scores in Rhenish German draught horses.

Horse farm-related source of variation	DF	Normal distribution		Multinomial distribution	
		F-value	*p*-Value	F-value	*p*-Value
Type of stable	4	3.98	0.0030	3.46	0.0085
Outdoor facilities for horses in winter	2	5.56	0.0041	3.17	0.0431
Daily hours horses in outdoor facilities in winter	2	4.22	0.0152	2.77	0.0636
Outdoor facilities for horses in summer	2	4.85	0.0083	5.09	0.0018
Bedding type	7	2.41	0.0199	2.19	0.0339
Time interval for cleaning out the stable	3	4.39	0.0046	4.18	0.0061

Type of roughage fed in winter months	4	2.43	0.0469	1.99	0.0953
Type of concentrate fed in winter months	1	2.82	0.0246	2.05	0.0859
Type of concentrate fed in summer months	4	2.48	0.0430	2.81	0.0251
Other additional feed types for horses	2	8.48	0.0002	5.83	0.0032
Type of hoof care	2	1.26	0.2841	1.20	0.3036
Length of hoof trimming intervals	4	5.76	0.0002	6.69	<0.0001
Type of work applications for horses	4	3.38	0.0097	3.50	0.0078
Daily hours of work with horses	2	3.54	0.0298	3.80	0.0230
Days per week with work with horses	4	1.74	0.1406	2.73	0.0280

Table S11. Least square mean estimates (LSM) for the fixed effects of horse farm-related effects with their standard errors (SE) and significant *p*-values (P-LSM) for differences between LSM of CPL-scores in Rhenish German draught horses using final model 2 with a normal distribution function.

Effect	Level and no of level		No of horses	**LSM ± SE**	**P-LSM**						
OUTS					2	3	4	5	6	7	8
	Paddock with open barn	1	34	2.78 ± 0.34			<0.0001				
	Pasture	2	357	1.86 ± 0.14			<0.0001				
	Pasture and paddock	3	92	1.82 ± 0.21			<0.0001				
	Unknown	4	10	0.76 ± 0.55							
BED											
	Barley straw	1	57	1.63 ± 0.26					0.014		0.016
	Rye straw	2	16	2.08 ± 0.39					0.009		0.031
	Wheat straw	3	127	1.50 ± 0.24					0.007		0.050
	Wood shavings	4	12	1.93 ± 0.37							0.013
	Barley + wheat straw	5	167	1.55 ± 0.26					0.011		0.034
	Rye + wheat straw	6	55	3.26 ± 0.53							0.001
	Any straw	7	28	1.61 ± 0.30					0.015		0.029
	Unknown	8	31	0.90 ± 0.29							
CLEAN											
	1-7 days	1	254	1.36 ± 0.17	0.002	0.001	0.016				
	2-4 weeks	2	145	1.82 ± 0.20							
	1-2 months	3	63	2.00 ± 0.22							
	>2 months	4	31	2.04 ± 0.29							
ROUW											
	Hay	1	198	1.71 ± 0.24	0.015						
	Hay + straw	2	89	1.32 ± 0.24		0.004	0.002				
	Hay + silage or haysilage	3	153	1.81 ± 0.26							
	Haysilage	4	39	1.97 ± 0.28							
	Unknown	5	14	1.22 ± 0.46							
CONW											
	Concentrates	1	377	2.01 ± 0.16	0.006						
	No concentrates	2	116	1.60 ± 0.21							
HOFT											

	4–8 weeks	1	147	1.92 ± 0.18			<0.0001				
	9–12 weeks	2	105	2.03 ± 0.19			<0.0001				
	13–16 weeks	3	85	2.00 ± 0.20			<0.0001				
	>16 weeks	4	26	1.10 ± 0.26							
	Unknown	5	130	1.98 ± 0.20				<0.0001			

Abbreviations: OUTS = Outdoor facilities for horses in summer, BED = Bedding type, CLEAN = Time interval for cleaning out the stable, ROUW = Type of roughage fed in winter months, CONW = Type of concentrate fed in winter months, HOFT = Length of hoof trimming intervals.

Table S12. Odds ratios (OR) for the fixed effects of horse farm-related effects with their 95% confidence intervals (95% CI) and significant *p*-values (C-Log) for differences between ORs of CPL-scores in Rhenish German draught horses using final model 2 with a multinomial distribution function.

Effect	Level and no of level		No of horses	**OR (95% CI)**	**C-Log**						
OUTS					2	3	4	5	6	7	8
	Paddock with open barn	1	34	1.0	0.0041		0.0036				
	Pasture	2	357	7.888 (1.935-32.157)							
	Pasture and paddock	3	92	5.897 (1.025-33.940)							
	Unknown	4	10	110.017 (4.667-999)							
BED											
	Barley straw	1	57	1.0					0.0016		0.0008
	Rye straw	2	16	0.107 (0.011-1.045)					0.0081		0.0005
	Wheat straw	3	127	1.296 (0.515-3.264)					0.0007		0.0032
	Wood shavings	4	12	0.748 (0.173-3.236)					0.0037		0.0045
	Barley + wheat straw	5	167	1.205 (0.509-2.854)					0.0012		0.0025
	Rye + wheat straw	6	55	0.006 (<0.001- 0.140)							<0.0001
	Any straw	7	28	1.287 (0.412-4.016)					0.0011		0.0050
	Unknown	8	31	11.018 (2.743-44.261)					<0.0001		
CLEAN											
	1-7 days	1	254	1.0	0.0005	0.0001	0.0019				
	2-4 weeks	2	145	0.286 (0.142-0.578)							
	1-2 months	3	63	0.286 (0.095-0.465)							
	>2 months	4	31	0.111 (0.028-0.442)							
ROUW											

	Hay	1	198	1.0	0.0192						
	Hay + straw	2	89	2.486 (1.161-5.325)		0.0217	0.0033				
	Hay + silage or haysilage	3	153	0.944 (0.475-1.879)							
	Haysilage	4	39	0.591 (0.263-1.329)							
	Unknown	5	14	0.315 (0.017-5.792)							
CONW											
	Concentrates	1	377	1.0	0.006						
	No concentrates	2	116	2.897 (1.478-5.679)							
HOFT											
	4–8 weeks	1	147	1.0			0.0002				
	9–12 weeks	2	105	0.808 (0.416-1.572)			<0.0001				
	13–16 weeks	3	85	0.931 (0.414- 2.096)			0.0012				
	>16 weeks	4	26	7.129 (2.561-19.847)				0.0014			
	Unknown	5	130	1.196 (0.600-2.382)							

Abbreviations: OUTS = Outdoor facilities for horses in summer, BED = Bedding type, CLEAN = Time interval for cleaning out the stable, ROUW = Type of roughage fed in winter months, CONW = Type of concentrate fed in winter months, HOFT = Length of hoof trimming intervals.

Table S13. Means and standard deviations for animal-related variables by males (n=80), geldings (n=55) and females (n=261) in Rhenish German draught horses aged >1 year.

Variate	Male	Gelding	Female
Height at withers (cm)	162.21 ± 6.20	164.35 ± 6.05	160.54 ± 5.52
Body length (cm)	171.74 ± 8.51	176.42 ± 9.21	177.75 ± 8.63
Chest circumference (cm)	202.54 ± 10.53	214.06 ± 9.98	213.61 ± 13.22
Skinfold thickness at the neck region (mm)	7.16 ± 1.66	6.47 ± 0.95	5.50 ± 1.12
**Front left limb**			
Cannon bone circumference (cm)	27.41 ± 3.57	27.07 ± 1.88	25.50 ± 2.86
Circumference coronary band (cm)	49.92 ± 2.91	50.50 ± 4.98	48.38 ± 4.62
Shore D hardness of hoof horn	59.33 ± 11.07	64.10 ± 10.26	62.82 ± 9.95
Length of the dorsal wall (cm)	10.80 ± 1.29	10.65 ± 1.17	10.38 ± 1.05
Length of the heel wall cm)	7.37 ± 1.92	7.46 ± 1.81	7.02 ± 1.80
Front hoof angle (°)	52.74 ± 4.58	52.76 ± 6.00	51.39 ± 5.67
**Hind right limb**			
Circumference of cannon bone (cm)	30.96 ± 3.45	30.53 ± 2.16	28.66 ± 2.27
Circumference coronary band (cm)	49.25 ± 3.96	49.55 ± 4.93	48.16 ± 3.76
Shore D hardness of hoof horn	57.75 ± 12.62	65.74 ± 11.68	63.92 ± 10.66
Length of the dorsal wall (cm)	10.18 ± 1.05	10.48 ± 1.25	10.09 ± 1.02
Length of the heel wall (cm)	6.83 ± 1.61	7.16 ± 1.51	6.93 ± 5.00
Front hoof angle (°)	55.09 ± 5.38	53.81 ± 4.52	52.12 ± 5.92

Table S14. *P*-Values of the generalized linear mixed model for each one animal-related variable as linear, quadratic and cubic covariate added to model 1 using a multinomial distribution for CPL-scores in Rhenish German draught horses.

Source of variation	Linear	Quadratic	Cubic
Height at withers	0.1581	0.1394	0.1246
Body length	0.4058	0.4494	0.4964
Chest circumference	0.9465	0.9686	0.9024
Skinfold thickness at the neck region	0.1055	0.0589	0.0713
**Front left limb**			
Cannon bone circumference	0.0016	0.0129	0.0506
Circumference of the coronary band	0.7531	0.8770	0.9677
Shore D hardness of hoof horn	0.5262	0.6014	0.6450
Length of the dorsal wall	0.5467	0.5238	0.5173
Length of the heel wall	0.1477	0.1139	0.1215
Front hoof angle	0.9555	0.8761	0.7820
**Hind right limb**			
Cannon bone circumference	<0.0001	0.0028	0.1151
Circumference of the coronary band	0.8204	0.8146	0.7811
Shore D hardness of hoof horn	0.1247	0.1047	0.1065
Length of the dorsal wall	0.4735	0.4269	0.4013
Length of the heel wall	0.4309	0.8417	0.9241
Front hoof angle	0.2843	0.3560	0.4008

Table S15. *P*-Values of the generalized linear mixed model for each one animal-related variable as linear, quadratic and cubic covariate by sex added to model 1 using a multinomial distribution for CPL-scores in Rhenish German draught horses.

**Source of variation**	**Linear**	**Quadratic**	**Cubic**
Height at withers	0.6888	0.6852	0.0440
Body length	0.9330	0.9317	0.4964
Chest circumference	0.6500	0.9686	0.6534
Skinfold thickness at the neck region	0.5083	0.5689	0.6901
**Front left limb**			
Cannon bone circumference	0.0179	0.0679	0.1579
Circumference of the coronary band	0.2515	0.2441	0.2338
Shore D hardness of hoof horn	0.1644	0.1841	0.1973
Length of the dorsal wall	0.8589	0.8369	0.8175
Length of the heel wall	0.0529	0.0545	0.0674
Front hoof angle	0.7996	0.7658	0.7278
**Hind right limb**			
Circumference of cannon bone	0.0004	0.0003	0.1151
Circumference of the coronary band	0.5079	0.5022	0.4897
Shore D hardness of hoof horn	0.3136	0.3026	0.3182
Length of the dorsal wall	0.5231	0.5398	0.5598
Length of the heel wall	0.4109	0.6205	0.6607
Front hoof angle	0.6792	0.7164	0.7250

Table S16. *P*-Values of the generalized linear mixed model for each one animal-related variable as linear, quadratic and cubic covariate and its interaction with age and sex (linear and quadratic for age) added to model 1 using a multinomial distribution for CPL-scores in Rhenish German draught horses.

**Source of variation**	**Linear**	**Quadratic**	**Cubic**
Height at withers	0.9789	0.9040	0.8256
Body length	0.6584	0.6951	0.7615
Chest circumference	0.6346	0.5862	0.5384
Skinfold thickness at the neck region	0.4268	0.4545	0.5769
**Front left limb**			
Cannon bone circumference	<0.0001	<0.0001	0.0004
Circumference of the coronary band	0.8511	0.9977	0.8249
Shore D hardness of hoof horn	0.6322	0.7076	0.7816
Length of the dorsal wall	0.8541	0.9094	0.9601
Length of the heel wall	0.1534	0.1148	0.1158
Front hoof angle	0.9213	0.9717	0.8705
**Hind right limb**			
Circumference of cannon bone	0.0027	0.0037	0.3604
Circumference of the coronary band	0.6309	0.6113	0.5740
Shore D hardness of hoof horn	0.1882	0.2132	0.2241
Length of the dorsal wall	0.6091	0.5783	0.5750
Length of the heel wall	0.3532	0.4844	0.5580
Front hoof angle	0.3912	0.3151	0.2949

Table S17. Results of the generalized linear mixed model (multinomial distribution with cumulative logit link function) with degrees of freedom (DF), F-values and *p*-values for cannon bone circumference as linear, quadratic and cubic covariate and as covariate interacting with sex and age (sex-age-covariate, linear and quadratic for age) added to model 1 using a multinomial distribution for CPL-scores of horses aged ≥3 year, ≥6 years, and ≥9 years in Rhenish German draught horses.

**Source of variation**	**DF**	**F-values for horses aged (years)**			***p*-Values for horses aged (years)**		
		**≥3**	**≥6**	**≥9**	**≥3**	**≥6**	**≥9**
**Front left**							
Linear	1	19.21	14.50	8.11	<0.0001	0.0002	0.0050
Quadratic	1	15.65	11.06	5.09	<0.0001	0.0010	0.0255
Cubic	1	11.96	8.12	3.56	0.0006	0.0048	0.0612
Sex-age-covariate							
Linear	3	4.51	4.51	1.63	0.0041	0.0043	0.1850
Quadratic	3	4.22	4.31	1.84	0.0060	0.0056	0.1430
**Hind right**							
Linear	1	9.91	10.55	11.59	0.0018	0.0014	0.0009
Quadratic	1	10.19	10.45	8.09	0.0016	0.0014	0.0051
Sex-age-covariate							
Linear	3	5.01	3.52	2.41	0.0021	0.0159	0.0695
Quadratic	3	4.45	3.06	2.06	0.0045	0.0294	0.1076
